# Testing use of payers to facilitate evidence-based practice adoption: protocol for a cluster-randomized trial

**DOI:** 10.1186/1748-5908-8-50

**Published:** 2013-05-10

**Authors:** Todd Molfenter, Jee-Seon Kim, Andrew Quanbeck, Terry Patel-Porter, Sandy Starr, Dennis McCarty

**Affiliations:** 1 Department of Industrial and Systems Engineering, University of Wisconsin-Madison, 1513 University Avenue, Madison, Wisconsin 53706, USA; 2 Ohio Dept. of Alcohol & Drug Addiction Services, 30 W. Spring Street, 6th Floor, Columbus, Ohio 43215-2256, USA; 3 Outcomes, and Research, Ohio Dept. of Alcohol & Drug Addiction Services, 30 W. Spring Street, 6th Floor, Columbus, Ohio 43215-2256, USA; 4 Oregon Health Sciences University, 3181 S.W. Sam Jackson Park Road, Portland, Oregon 97239-3098, USA

**Keywords:** Evidence-based practice implementation, Buprenorphine, Addiction treatment, Innovation

## Abstract

**Background:**

More effective methods are needed to implement evidence-based findings into practice. The Advancing Recovery Framework offers a multi-level approach to evidence-based practice implementation by aligning purchasing and regulatory policies at the payer level with organizational change strategies at the organizational level.

**Methods:**

The Advancing Recovery Buprenorphine Implementation Study is a cluster-randomized controlled trial designed to increase use of the evidence-based practice buprenorphine medication to treat opiate addiction. Ohio Alcohol, Drug Addiction, and Mental Health Services Boards (ADAMHS), who are payers, and their addiction treatment organizations were recruited for a trial to assess the effects of payer and treatment organization changes (using the Advancing Recovery Framework) versus treatment organization changes alone on the use of buprenorphine. A matched-pair randomization, based on county characteristics, was applied, resulting in seven county ADAMHS boards and twenty-five treatment organizations in each arm. Opioid dependent patients are nested within cluster (treatment organization), and treatment organization clusters are nested within ADAMHS county board. The primary outcome is the percentage of individuals with an opioid dependence diagnosis who use buprenorphine during the 24-month intervention period and the 12-month sustainability period. The trial is currently in the baseline data collection stage.

**Discussion:**

Although addiction treatment providers are under increasing pressure to implement evidence-based practices that have been proven to improve patient outcomes, adoption of these practices lags, compared to other areas of healthcare. Reasons frequently cited for the slow adoption of EBPs in addiction treatment include, regulatory issues, staff, or client resistance and lack of resources. Yet the way addiction treatment is funded, the payer’s role—has not received a lot of attention in research on EBP adoption.

This research is unique because it investigates the role of payers in evidence-based practice implementation using a randomized controlled design instead of case examples. The testing of the Advancing Recovery Framework is designed to broaden the understanding of the impact payers have on evidence-based practice (EBP) adoption.

**Trial registration:**

http://NCT01702142 (ClinicalTrials.gov registry, USA)

## Background

The use of evidence-based clinical practices in health care is suboptimal [1,2]. The addiction treatment field adopts evidence-based practices (EBPs) at an even lower rate than general health care [3-5]. For example, use of medication-assisted therapy for addiction disorders is not standard practice, despite the evidence that medication improves clinical, societal and financial outcomes through increased abstinence rates [6], reduced relapse rates [7], and reduced criminal costs [8]. A survey of 354 private addiction treatment centers reported that 34 percent of programs provided medication for opioid dependence and 24% used medication for treatment of alcohol dependence [9]. Within treatment programs that do offer medication, only 40% of the treatment plans for opioid dependence included medication [10].

Buprenorphine received Food and Drug Administration approval for treating opioid dependence in 2002 but remains underutilized. Less than one-fifth of patients with an opioid dependence diagnosis are offered buprenorphine [11,12]. Its adoption has been impeded by behavioral resistance, lack of or low reimbursement, insufficient access to personnel needed to prescribe the medication, licensure and contractual rules that prohibit the use of any pharmacotherapies in some settings, and insufficient organizational systems to encourage its use. Buprenorphine was selected as a candidate for EBP implementation and dissemination research because the broad set of behavioral and systemic barriers that limit its use incorporates factors that affect other EBPs with poor use rates.

Payer policy and organizational interventions can address barriers to the adoption of medication (and evidence-based practices more generally) [13,14]. Policy makers and local organizations collaborate to disseminate school reform, alternative energy use, and third-world agricultural practices [15,16]. Few trials in healthcare have tested the impact of aligning payer policy and treatment organization interventions to enhance implementation of treatment innovations [17]. However, from 2006 through 2010, NIATx (formerly the Network for the Improvement of Addiction Treatment), a process improvement research center based at the University of Wisconsin–Madison, conducted a demonstration project titled ‘Advancing Recovery: State and Treatment Organization Partnerships for Quality Addiction Care’. Funded by the Robert Wood Johnson Foundation, Advancing Recovery promoted the use of evidence-based practices such as medication-assisted treatment by building relationships between treatment organizations and the payers that fund their services. These partnerships made system changes to regulatory, clinical and administrative practices to increase the implementation of EBPs [18]. Advancing Recovery involved payer-treatment organization partnerships in 12 states, but was not a randomized controlled trial. The Advancing Recovery demonstration project did develop a framework for systems change to be tested in this randomized trial, calling it the Advancing Recovery Framework.

### Objectives

The primary research question is to determine if the Advancing Recovery Framework increases the use of buprenorphine in addiction treatment organizations. The secondary research question is to determine if county characteristics (*e.g*., percentage of opioid abusers, existing addiction treatment medication availability, and payment policy) and organizational characteristics (*e.g.*, organization size, access to physicians, number of nurses, percentage of clinicians with graduate degrees, and patient demographic characteristics) can moderate the delivery of buprenorphine.

## Methods

### Trial design

**Figure 1:**
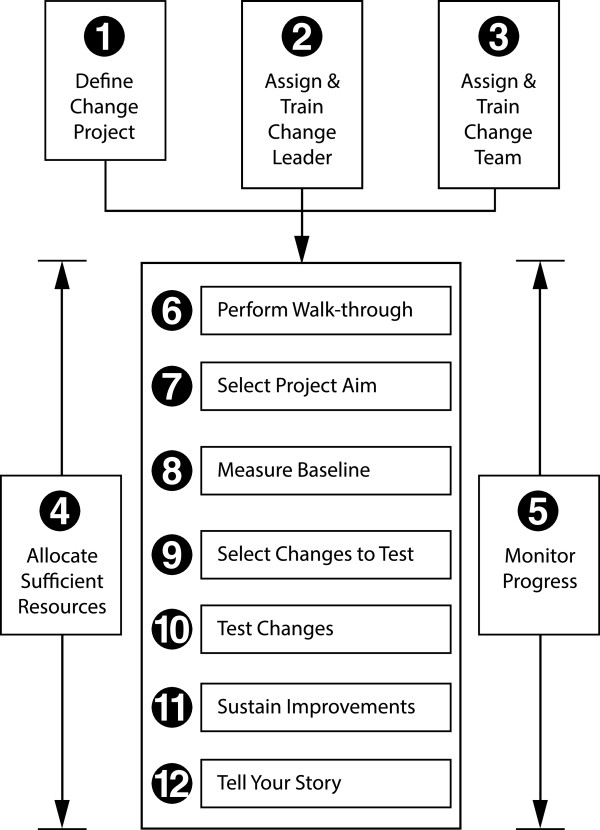
The NIATx organizational change model.

The participating ADAMHS boards have been randomized into two study conditions. In the Control Arm (Arm 1), participants will use the NIATx model for organizational change only. In Arm 2, participants will use the Advancing Recovery Framework that includes the payer intervention and NIATx models. Payers will not play a direct role in the control arm. The NIATx organizational change model (Figure 1) was chosen as the control condition because research exists that suggests organizational change models alone may be sufficient for EBP implementation [19,20]. A cluster randomized controlled design was used to study the effects that payers (or county ADAMHS boards) and treatment organizations have on buprenorphine adoption and also to control for ‘contamination’ across the treatment and control arms. A 1:1 allocation ratio of counties was utilized in the randomization.

### Participants

**Figure 2:**
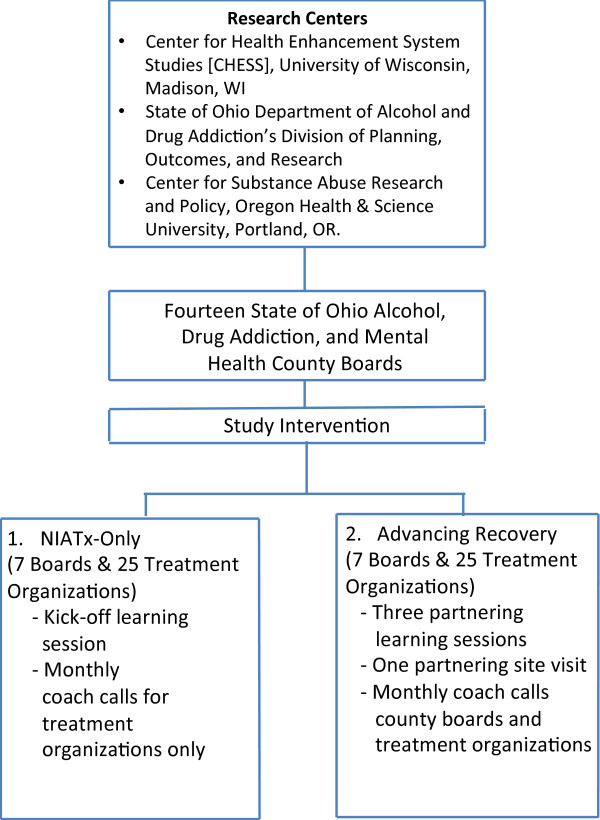
Consort Diagram (Enrollment & Intervention Phases).

Ohio Alcohol, Drug Addiction and Mental Health Boards (ADAMHS) coordinate the public behavioral health system in Ohio. Each board covers one to five of Ohio’s 88 total counties and disperses Substance Abuse and Prevention Treatment (SAPT) block grant funds, as well as funds from local levies directed to addiction services. These Boards are responsible for 55% of addiction treatment services funding and often have responsibility for the quality and cost effectiveness of addiction treatment services within their jurisdictions [21]. The boards contract with provider organizations to deliver addiction and mental health treatment services. All 46 of Ohio’s ADAMHS boards were eligible for the study. The ADAMHS boards were recruited through an invitation from the Ohio Association of County Behavioral Health Authorities from May to September, 2012. The study team also actively recruited ADAMHS boards needed to meet the study’s matching criteria. A total of 14 boards representing 23 counties agreed to participate (Figure 2). Within each board jurisdiction, treatment agencies with 75 or more annual admission were invited to participate in the study. Fifty treatment agencies volunteered. Two to eight treatment agencies are participating per board.

### Interventions

The Advancing Recovery Framework builds upon the multi-organizational learning collaborative method used in the NIATx 200 national trial to implement process improvement strategies in over 200 treatment organizations [22,23]. The Advancing Recovery Framework delivers technical assistance through learning sessions and coaching. Learning sessions are face-to-face meetings that occur once in the NIATx-only arm and three times in the Advancing Recovery arm to facilitate partnering between the ADAMHS boards and their treatment organizations. In these sessions, change teams from the ADAMHS board area (Arm 2 only) and each of the treatment organizations convene in face-to-face meetings to support and encourage each other. Outside experts offer advice on changes to make and ways to make them. Coaching assigns an expert in medication-assisted treatment adoption and process improvement to help the ADAMHS board and its treatment organizations make, sustain and spread buprenorphine adoption efforts. Coaches help the ADAMHS boards’ counties in Arm 2, and organizations in both arms think through key issues, broker relationships with other organizations, offer process improvement training, and suggest changes to make and how to do so. Participants in both arms receive coaching through monthly group teleconference calls, through e-mail, and at in-person learning sessions.

The payer intervention portion of the Advancing Recovery Framework has four phases: ‘Pre-Work for Leadership’ identifies the individuals who will lead and execute the change project; ‘Preparing the Change Leader & Team’ provides training in change management techniques; ‘Planning’ uses a five-lever policy analysis to catalog barriers to buprenorphine use and pilot tests potential tactics to remedy; and ‘Implementation’ takes tactics to apply from the planning phase, implements them, and focuses on sustaining the gains. During the learning sessions and monthly coach calls between the payer and treatment organizations, the payer has the opportunity to partner and collaborate with treatment organizations. Table 1 outlines the steps, tools, and training methods for each phase of the payer intervention model.

**Table 1:** Payer intervention model of the advancing recovery framework (applied in Arm 2 only)

**Phase**^**1**^	**Steps**	**Tools**	**Training method(s)**
**Pre-work for Leadership**	To be completed as part of Executive Briefing by the Executive Director (or designee)	Executive sponsor (County Director) personal responsibilities checklist	Coach call to Executive Sponsor to review primer for Executive Sponsor
	1. Identify an Executive Sponsor for payer portion of the project		Change leader training on how to apply and interpret the 5 Levers Barriers Assessment
	2. Develop the strategic aim for buprenorphine adoption.		
	3. Select change leader and team members who are committed and make sure they have adequate time to participate in the project.		
**Preparing the Change Leader & Team**	4. Change Leader & Team Training on organizational and policy change techniques.	NIATx Workbook (Change Leader Training Guide – Administrative Version)	Provided in Learning Session 1 and reinforced by coaches throughout
**Planning (in conjunction with the treatment organization community)**	5. Conduct the 5 Levers Barriers Assessment. (Customer Impact, Financial & Purchasing, Regulatory & Policy, Operational, & Inter-organizational) to identify list of potential barriers to remove	5-Levers Policy-Barriers Assessment	Provided in Learning Session 1 and reinforced by coaches throughout
	6. Develop tactics to apply	Implementation charter form	
	7. Pilot test *tactics*		
**Implementation**	8. Develop list of *tactics* to implement (based on planning)	Implementation charter form	Change leader training in Learning Sessions on the implementation and sustainability process
	9. Assign a process owner, and timeline with deliverables for each *tactic*	Apply payer-based sustainability assessment form	
	10. Implement changes		
	11. Sustain gains		

The five levers described in the planning phase provide a mechanism for payers to identify systemic barriers to EBP use and promote change. The ‘customer impact analysis’ examines how the EBP benefits the customer, affects the number or type (*i.e.*, demographic) of customers involved in treatment, and which customers are needed to advocate for the change. A ‘financing and purchasing analysis’ determines how financing, reimbursement and payment provide incentives for EBP use. The ‘regulatory and policy analysis’ uncovers regulatory barriers. The ‘operations analysis’ assesses the training and skill-building payers and treatment organizations need for EBP implementation. Finally, an ‘inter-organizational analysis’ identifies what other organizations need to be involved to remove regulatory or financing barriers.

### Timeline

During project months 1 to 5, the study team will identify and recruit counties (through the Ohio Association of County Behavioral Health Authorities) and treatment organizations. In months 6 to 11, the study team will collect baseline data on buprenorphine use rates, conduct buprenorphine training workshops for treatment organizations, and update NIATx training materials to reflect the study conditions. In months 12 to 36, the study team will implement the study arm interventions and collect outcome data. During months 37 to 50, the study team will collect quantitative data on the sustainability of the changes. Data analysis, publication development, and dissemination of findings will occur at several points, but primarily during months 51 to 60.

### Sample size

To determine sample size, we fit a linear mixed-effects model to the monthly results for the performance measure of percent opioid dependent patients receiving buprenorphine from each of the participating organizations and estimate the ‘payer-treatment organization’ effect. A mixed-effect model was selected instead of conventional regression models because, first, the mean outcomes for a given treatment organization are expected to be correlated from month to month. A mixed-effect model allows for auto-correlated error terms. Second, a number of unobservable treatment organization characteristics are likely to influence the key measures. Random effects at the treatment organization level can be employed within the mixed-effect model to capture the correlation introduced by such characteristics. Proper reflection of auto-correlation and random effects allows for more reliable estimates of the fixed effects, starting in months 6 to 11 (for baseline), then during months 12 to 36 (intervention stage), and through months 37 to 50 (follow-up stage). The power of the study design is determined by the anticipated standardized effect size based on effects experienced in the Advancing Recovery pilot project. The Advancing Recovery pilot data found that the payer-treatment organization intervention increased buprenorphine use rates from 6% to 43%, with an estimated standard deviation of 23.5% based on a sample size of 1,016. This yields an effect size of 0.935. Intraclass correlation (ICC) among counties affects the power of cluster-randomized trials [24]. An estimate of ICC is around 0.05. For this study, a dropout rate of 20% is being proposed, giving a total of 40 organizations available for analysis. With a total of 40 treatment organizations, each with an average of 91 patients, the study will achieve at least a power of 0.82 with a one-tailed type I error rate of 0.05. Power and sample size calculations were performed using Optimal Design Software by Raudenbush (Version 3.01) [25]*.*

### Randomization & implementation

Matched-pair blocks were created on the basis of the county characteristics of: percentage of opiate admissions; population covered; and ratio of opiate addiction treatment admissions to physicians licensed to prescribe buprenorphine [26]. The cut-off criteria used for matching, based on the median measures from the 2011 Ohio Department of Alcohol and Drug Addiction Services treatment organization dataset, were: 20% opiate admissions (above/below); 200,00 people covered (above/below); and 50 treatment admissions per physicians in the county registered to prescribe buprenorphine per annum (above/below). As a result, seven county board matched-pairs were identified. A random number generator was utilized within the seven county board pairs to assign organizations into one of the two study conditions. Study staff completed enrollment and received consent approval from the county boards as well as providers. No visible concealment was applied, and blinding of participants and researchers was considered infeasible due to logistical reasons.

### Data analysis

Because the number of treatment organizations within an ADAMHS Board is small (n = 2 to 8) we intend to use an optimal design in which treatment arms are allocated following a multi-site cluster randomization procedure [27,28]. The initial exploratory analyses of both outcomes and covariates will assess standard summary statistics and graphical presentations. Scatter plots and correlation analyses identify possible associations between outcome and predictor variables. The analysis will represent the multi-site cluster randomized trial as a mixed-effects model; percentage of buprenorphine use by opioid dependent patients is nested within cluster (treatment organization); and clusters are nested within site (ADAMHS board). In this mixed-effects model, there will be random effects due to county board and organization; fixed effect due to study arm and time. For the percent of opioid admissions receiving buprenorphine, a logistic regression analysis will be performed. We will examine the Advancing Recovery effect using a separate model at each time point and in an omnibus growth curve model that includes the main effects of Advancing Recovery and Time as well as the interaction between Advancing Recovery and Time. The percent of opioid patients receiving buprenorphine will be measured monthly for each organization, and it is expected that these values will be correlated over time. Therefore, instead of assuming an independence (*i.e*., zero correlation) or a compound symmetry covariance structure (*i.e*., a constant correlation regardless of the proximity of measurement time points), we will allow errors to be first-order auto-correlated, denoted as AR (1). This can be done by allowing an additional parameter in the mixed-effects model that represents the correlation between the two most adjacent measures of Opioid Patients Receiving Buprenorphine. This correlation is reduced exponentially as the measures become further apart.

For the ADAMHS board and organizational covariates, at each time point we will assess moderation by including each potential moderator as an independent variable and as an interaction with the Advancing Recovery effect. Statistical significance of the interaction effect will indicate the presence of moderation. The estimate for the effect size of moderation, which is cross-level interaction, is between 0.17 and 0.19.

### Ethics

The study received approval from the institutional review boards at the University of Wisconsin–Madison, Ohio Department of Health’s Human Subjects Institutional Review Board, and Oregon Health and Science University.

### Trial status

The recruitment phase of the trial has just been completed and baseline data collection has begun.

## Discussion

Conceptual multi-level models of EBP adoption are present in the literature and are based on correlates of EBP adoption discovered at the larger systems/environment, organizational, and individual levels [13,17,29]. These models identify which factors EBP implementation efforts should attempt to influence at the different levels in the healthcare system, but do not address how to change these factors. Accordingly, this research represents a movement from correlate identification to implementation model testing. This progression is described in McGovern’s ‘Implementation Research Flow’ paradigm, which explains how the purpose of implementation models is to positively influence the implementation and maintenance trajectories of the EBP adoption [30].

At present, the implementation research on how administrative practices at the payer level promote use of clinical practices at the treatment organization level primarily relies on case examples. The described research protocol has the unique aspect of randomizing by payer. The use of implementation research randomizing by county has been limited [31,32], and no instances of randomization at the state level were found. Research studying use of payer administrative practices to promote clinical evidence-based practice implementation should begin to use randomized trials to augment and validate findings found in payer case-examples.

This trial is taking lessons from past implementation research through case examples from the Advancing Recovery pilot [33-39], and packages them into an implementation model [18]. The research trial testing the Advancing Recovery Framework will build on several implementation research questions: What are the roles of payers with planning and stakeholder engagement in the provision of EBPs? What policy levers have an impact on EBP implementation and sustainability rate? What are the synergistic effects of having multiple levels focused on implementing a targeted EBP? What is the role of leadership at the payer and provider levels in the broader use of EBPs? What training and technical assistance offerings in the learning collaborative model are most helpful in a multi-level design?

Ohio was selected as a research site because it was considered to be a representative governmental healthcare environment to test this implementation model. A total of 34% of the states in the United States have county or region boards to distribute SAPT block grant funds. Each Ohio ADAMHS board represents a unique payer environment. The state has a mix of urban and rural counties. Within these counties, buprenorphine use varies from 7% to 58%. Limitations could be present due to the research environment. This trial addresses governmental payers securing healthcare services. Governmental healthcare is a considerable enterprise, where an effective framework for evidence-based practice implementation could prove to be extremely helpful. While this trial may not be generalizable to governmental payers for other community services, the Advancing Recovery framework could give these sectors interventions to consider in their efforts to improve services.

## Conclusion

In conclusion, this trial will contribute to the evolving implementation science field over the next five years. Future results of this multi-level approach will be published as they become available.

## Abbreviations

ADAMHS: Alcohol, Drug Addiction and Mental Health Services; EBP: Evidence-based practice; ICC: Intraclass correlation; NIATx: Network for the Improvement of Addiction Treatment; SAPT: Substance Abuse and Prevention Treatment.

## Competing interests

The authors declare no competing interests.

## Authors’ contributions

All authors made substantial contributions to the study design as well as the development and editing of the manuscript. All authors read and approved the final manuscript.
